# Pregnancy Complications and Neonatal Outcomes in Multiple Pregnancies: A Comparison between Assisted Reproductive Techniques and Spontaneous Conception

**DOI:** 10.22074/ijfs.2015.4175

**Published:** 2015-02-07

**Authors:** Mahbod Kaveh, Mahsa Ghajarzadeh, Fatemeh Davari Tanha, Fatemeh Nayeri, Zahra Keramati, Mamak Shariat, Azadeh Ghaheri

**Affiliations:** 1 Women Hospital, Tehran University of Medical Sciences, Tehran, Iran; 2 Tehran University of Medical Sciences, Tehran, Iran; 3 Department of Obstetrics and Genecology, Women Hospital, Tehran University of Medical Sciences, Tehran, Iran; 4 Imam Khomeini Hospital, Tehran University of Medical Sciences, Tehran, Iran; 5 Maternal, Fetal and Neonatal Research Center-Breastfeeding Research Center, Tehran University of Medical Sciences, Tehran, Iran; 6 Department of Epidemiology and Reproductive Health at Reproductive Epidemiology Research Center, Royan Institute for Reproductive Biomedicine, ACECR, Tehran, Iran

**Keywords:** Assisted Reproductive Technology, Multiple Pregnancies, Outcome, Pregnancy Complications

## Abstract

**Background:**

This study compared neonatal outcome and maternal complications in multiple pregnancies after assisted reproductive technologies (ART) to spontaneous pregnancies.

**Materials and Methods:**

In this cross-sectional study, we reviewed medical records of 190 multiple pregnancies and births conceived by ART or spontaneous conceptions between 2004 and 2009 in Women Hospital. Obstetric history and outcomes were recorded and compared between these two groups. SPSS version 13 was used for data analysis. The results were analyzed using student’s t test, chi square and logistic regression (p<0.05).

**Results:**

There were 106 deliveries from spontaneous conceptions and 84 that resulted from ART. Parity history and mode of delivery significantly differed between the two groups (p<0.001). The ART group had significantly higher preterm labor and premature rupture of membranes (PROM) whereas pregnanc-induced hypertension (PIH) was higher in the spontaneous group (p=0.01). Newborn intensive care unit (NICU) admission, duration of hospitalization, still birth and low gestational age were significantly higher in the ART group while neonatal jaundice was higher in the spontaneous group. Logistic regression analysis by considering neonatal complications as the dependent variable showed that respiratory distress syndrome (RDS), NICU admission and Apgar score were independent predictors for neonatal complications.

**Conclusion:**

Obstetric and neonatal outcomes must be considered in multiple pregnancies conceived by ART.

## Introduction

Assisted reproductive technologies (ART) are used worldwide for infertility treatment associated with multiple pregnancies (1). Although ART is a technique for infertile couples to have children, it is associated with numerous risks such as congenital malformations, chromosomal aberrations and multiple pregnancies (2). To increase the success of ART, more than one embryo is transferred in a cycle which increases the risk of multiple pregnancies and births. Twin deliveries are reported at 20-30% in the US and Europe; triple deliveries range from 2.2-6% in couples who have undergone ART cycles. This rate is 1-2% in spontaneous conceptions (3, 4).

Premature births, low birth weight, high blood pressure, and small-for-gestational-age (SGA) were reported at higher rates in infants conceived with ART compared to spontaneous conceptions which increased prenatal mortality and morbidity (5). In a study by Martin et al., mean gestational age of 35 weeks for twin and 32 weeks for triplet neonates was reported (6).

Previous studies have suggested that ART infants have a more deprived perinatal outcome that is associated with increased neonatal intensive care unit (NICU) admissions and longer hospitalizations. Consequently, neonatal deaths have been reported to be approximately 56 per 1000 live twin births and 176 per 1000 live births in triplet or higher deliveries (7). The infant death rate is approximately 72 per 1000 live twin births and 204 per 1000 in triplets or higher deliveries (8).

On the other hand, obstetric outcomes after ART cycles have been shown to be related to higher rates of pregnancy-induced hypertension (PIH), vaginal bleeding, premature rupture of membranes (PROM), elective cesarean section (C/S) and still births (7, 9).

The goal of this study was to compare neonatal outcome and maternal complications in multiple pregnancies after ART to spontaneous conceptions.

## Materials and Methods

In this cross-sectional study, we reviewed the medical records of multiple pregnancies and births conceived by ART or spontaneous conceptions that occurred in Women Hospital, an affiliated hospital of Tehran University of Medical Sciences, between 2004 and 2009. There were 190 multiple deliveries from which 84 used ART cycles and 106 were from spontaneous conceptions. Obstetric history and outcomes such as gestational age, icter, preterm birth, perinatal mortality, PROM, maternal age, delivery mode [normal vaginal delivery (NVD) or C/S], parity, anemia, congenital malformations, maternal death, PIH, pre- and post-natal bleeding, Apgar score, respiratory distress syndrome (RDS), NICU admission, and maternal and neonatal duration of hospitalization were recorded for each group.

SPSS version 13 was used for data analysis. Data are presented as mean ± SD and the student’s t test for quantitative variables, frequencies and percentages and the chi-square test for qualitative variables. Logistic regression was applied for odds ratio (OR) calculation and comparison between the ART and spontaneous groups as dependent variables that used maternal and neonatal complications. P values under 0.05 were considered statistically significant.

## Results

We reviewed the medical records of 190 multiple deliveries. Of these, 106 were spontaneous conceptions and 84 were conceived by ART. Table 1 shows the mothers’ obstetric histories in the ART and spontaneous groups.

 The mean ± standard deviation for gestational age was 34.6 ± 3.4 weeks for twin deliveries, 31.3 ± 3.9 weeks for triplets and 30.6 ± 4.9 weeks for quadruplets. Table 2 compares maternal prenatal complications between the ART and spontaneous groups. One maternal death occurred in the spontaneous group because of septicemia as a complication of fetal death. Blood transfusions were given to two cases of post-natal bleeding. The results obtained from the preliminary analysis of neonatal complications in the ART and spontaneous groups are presented in table 3.

We used logistic regression and considered preterm birth as a confounding variable, an Apgar score <7, RDS and NICU remained in the neonatal complication model and PROM and pregnancy induced hypertension (PIH) remained in the maternal complication model. The adjusted OR (AOR) for PROM was 0.2 which indicated that with a unit increase in PROM the chance of being in the ART group was five times the chance of being in the spontaneous group (Tables4, 5).

**Table 1:** Descriptive statistics for maternal variables in the assisted reproductive technology (ART) and spontaneous groups


Variables	Spontaneous	ART	P value

**Maternal age (Y)**	28.18 ± 6.06	27.2 ± 4.2	0.1
**Parity history**			
Nulliparous	60 (56.6%)	68 (81%)	<0.001*
Multiparous	46 (43.4%)	16 (19%)	
**Delivery mode**			
NVD	2 (1.9%)	16 (19%)	<0.001*
C/S	104 (98.1%)	68 (81%)	


The results are reported as mean ± SD and frequency (percentage). NVD; Normal vaginal delivery, C/S; Cesarean section and *; P<0/05 is statistically significant.

**Table 2:** Maternal prenatal complications in the assisted reproductive technology (ART) and spontaneous groups


Variables	Spontaneous	ART	P value

**Preterm labor**	66 (62.3%)	66 (78.6%)	0.01*
**Anemia**	5 (4.7%)	8(9.5%)	0.1
**PROM**	22 (20.7%)	29 (34.5%)	0.03*
**Maternal death**	1 (0.9%)	0	-
**Antenatal bleeding**	3 (2.8%)	5(6%)	0.4
**Post-partum bleeding**	3 (2.8%)	0	-
**PIH**	24 (22.6%)	8(9.5%)	0.01*
**Duration of hospitalization**			
**<7 days**	94 (88.7%)	66 (78.6%)	0.05*
**≥7 days**	12 (11.3%)	18 (21.4%)	


The results are reported as frequency (percentage). NICU; Newborn intensive care unit, RDS; Respiratory distress syndrome, PROM; Premature rupture of membranes, PIH; Pregnancy-induced hypertension and *; P<0/05 is significant.

**Table 3:** Neonatal complications in the assisted reproductive technology (ART) and spontaneous groups


Variables	Spontaneous	ART	P value

**Gestational age (weeks)**			
**20-28**	5 (4.7%)	6 (7.1%)	
**29-32**	8 (7.5%)	13(15.4%)	<0.001*
**33-36**	51(48.6%)	49(58.3%)	
**≥37**	42(39.2%)	16(19%)	
**NICU admission**	33(31.1%)	50(59.5%)	<0.001*
**Birth weight discordance**	15(14.1%)	18(21.4%)	0.2
**RDS**	31(29.2%)	20(23.8%)	<0.001*
**Neonatal jaundice**	53(50%)	32(38%)	<0.001*
**First min Apgar score**			
**<7**	16(15%)	13(15.4%)	0.003*
**≥7**	90(84.9%)	71(84.5%)	
**Sepsis**	21(19.8%)	30(35.7%)	<0.001*
**Duration of hospitalization (days)**			
**<7**	85(80.1%)	58(69%)	0.002*
**≥7**	21(19.8%)	26(30.9%)	
**Congenital malformations**	9 (8.4%)	15(17.8%)	0.1
**Still births**	16(15%)	28(33.3%)	0.03*


The results are reported as frequency (percentage). NICU; Newborn intensive care unit, RDS; Respiratory distress syndrome and *; P<0/05 is significant.

**Table 4:** Adjusted odds ratios (AOR) for neonatal complication model


Variables	AOR	95% CI	P value

**Low birth weight**	1.4	0.6-3	0.3
**Icter**	0.6	0.38-10.5	0.12
**Apgar <7**	2.1	1-4.3	0.03*
**Sepsis**	1.6	0.6-4	0.2
**RDS**	2.1	1.2-3.8	0.01*
**NICU**	2.3	1.3-4.1	0.004*


NICU; Neonatal intensive care unit, RDS; Respiratory distress syndrome and *; P<0/05 is significant. The OR is adjusted for "preterm birth".

**Table 5:** Adjusted odds ratios (AOR) for maternal complication model


Variables	AOR	95% CI	P value

**PROM**	0.2	0.08-0.7	0.01*
**PIH**	7.8	4-15.3	0.001*
**Anemia**	2.1	0.7-6	0.17


PROM; Premature rupture of membranes, PIH; Pregnancy induced hypertension and *; P<0/05 is significant. The OR is adjusted for "preterm birth".

## Discussion

Our study showed that twins conceived by ART had a greater risk of adverse perinatal outcome as well as low gestational age, RDS, neonatal jaundice, still birth, sepsis, low Apgar score, longer duration of hospitalization and more NICU admissions. Our results were consistent with previous findings which showed that twins born with ART methods had longer birth admissions and increased possibility for NICU admissions (7). Pinborg et al. compared twins of ART methods with twins by spontaneously conceived pregnancies and found higher levels of preterm (<37 weeks) births in the ART group (10).

Multiple pregnancies are the main problem of ART treatment in infertile couples. Multiple pregnancies are associated with four to ten times greater perinatal mortality compared to singleton gestations. A total of 12.2% of all preterm infants, 15.4% of all neonatal deaths, and 9.5% of fetal deaths have been related to twin deliveries (11).Filicori et al. (12) suggested that low and very low birth weights were more likely to increase in infants conceived with ART than spontaneous conceptions. It has been proposed that infertility itself increases risk of preterm birth and low birth weight due to investigation of worse prenatal and obstetric complications of singleton pregnancies after ART (12, 13).

The present study had higher prevalence of preterm labor and PROM in the ART group compared to the spontaneous group. It has been reported that preterm labor occurs in near half of twin pregnancies and likely to occur four times greater among multiple than singleton gestations (14, 15). Our findings supported the results reported by Tan et al. (16) in which preterm labor occurred in 58% of the ART group compared with 52% in the control group. In contrast, Zaib-un-Nisa et al. (17) found preterm labor in 42% of ART twin deliveries compared with 51% spontaneous twin deliveries.

Our results showed a lower rate of pregnancy-induced hypertension and postpartum bleeding in women who underwent ART compared to the spontaneous group. These findings contradicted previous investigations. Oleszczuk et al. reported no significant difference in pregnancy-induced hypertension and vaginal bleeding in assisted conceptions compared with the spontaneous group (8). In other studies multiple pregnancies have been related to increased risk of PIH during pregnancy, however this increase was not statistically significant (18, 19).

Increased concern about maternal and neonatal well-being makes women carrying twin pregnancies conceived by ART more likely to undergo elective C/S (20). Older maternal age and first pregnancy in higher age of the ART group reported in previous studies (17, 21). We found no significant difference between maternal ages of both groups; however women who underwent ART were more frequently primiparous.

In the current study, RDS, NICU admission and Apgar score were independent predictors for neonatal complications. Multiple pregnancies are higher in assisted pregnancies due to various factors such as ovulation induction and multiple embryo transfers. Hence the rate of maternal mortality and perinatal mortality and morbidity are also higher. The rate of complication arises along with the increase in numbers of fetuses. A perinatal mortality rate of 4.2 for single and 16.6 for multiple pregnancies has been recorded in a study in 2009 (22).

Higher rates of chromosomal abnormalities, preterm birth, growth retardation, cerebral palsy, NICU admissions and death in one year are neonatal complications attributed to ART induced pregnancies. Bleeding during pregnancy and the post-partum period, high blood pressure, preeclampsia and anemia are higher in mothers with ART induced pregnancies (1, 22).

## Conclusion

The both obstetric and neonatal outcomes must be considered in ART multiple pregnancies.
